# The Role of Flexible Loops in Folding, Trafficking and Activity of Equilibrative Nucleoside Transporters

**DOI:** 10.1371/journal.pone.0136779

**Published:** 2015-09-25

**Authors:** Jaya Aseervatham, Lucky Tran, Khaled Machaca, Olga Boudker

**Affiliations:** 1 Department of Physiology and Biophysics, Weill Cornell Medical College, New York, United States of America; 2 Department of Physiology and Biophysics, Weill Cornell Medical College in Qatar, Qatar foundation, Education City, Doha, Qatar; University of Minho, PORTUGAL

## Abstract

Equilibrative nucleoside transporters (ENTs) are integral membrane proteins, which reside in plasma membranes of all eukaryotic cells and mediate thermodynamically downhill transport of nucleosides. This process is essential for nucleoside recycling, and also plays a key role in terminating adenosine-mediated cellular signaling. Furthermore, ENTs mediate the uptake of many drugs, including anticancer and antiviral nucleoside analogues. The structure and mechanism, by which ENTs catalyze trans-membrane transport of their substrates, remain unknown. To identify the core of the transporter needed for stability, activity, and for its correct trafficking to the plasma membrane, we have expressed human ENT deletion mutants in *Xenopus laevis* oocytes and determined their localization, transport properties and susceptibility to inhibition. We found that the carboxyl terminal trans-membrane segments are essential for correct protein folding and trafficking. In contrast, the soluble extracellular and intracellular loops appear to be dispensable, and must be involved in the fine-tuning of transport regulation.

## Introduction

**Fig 1:**
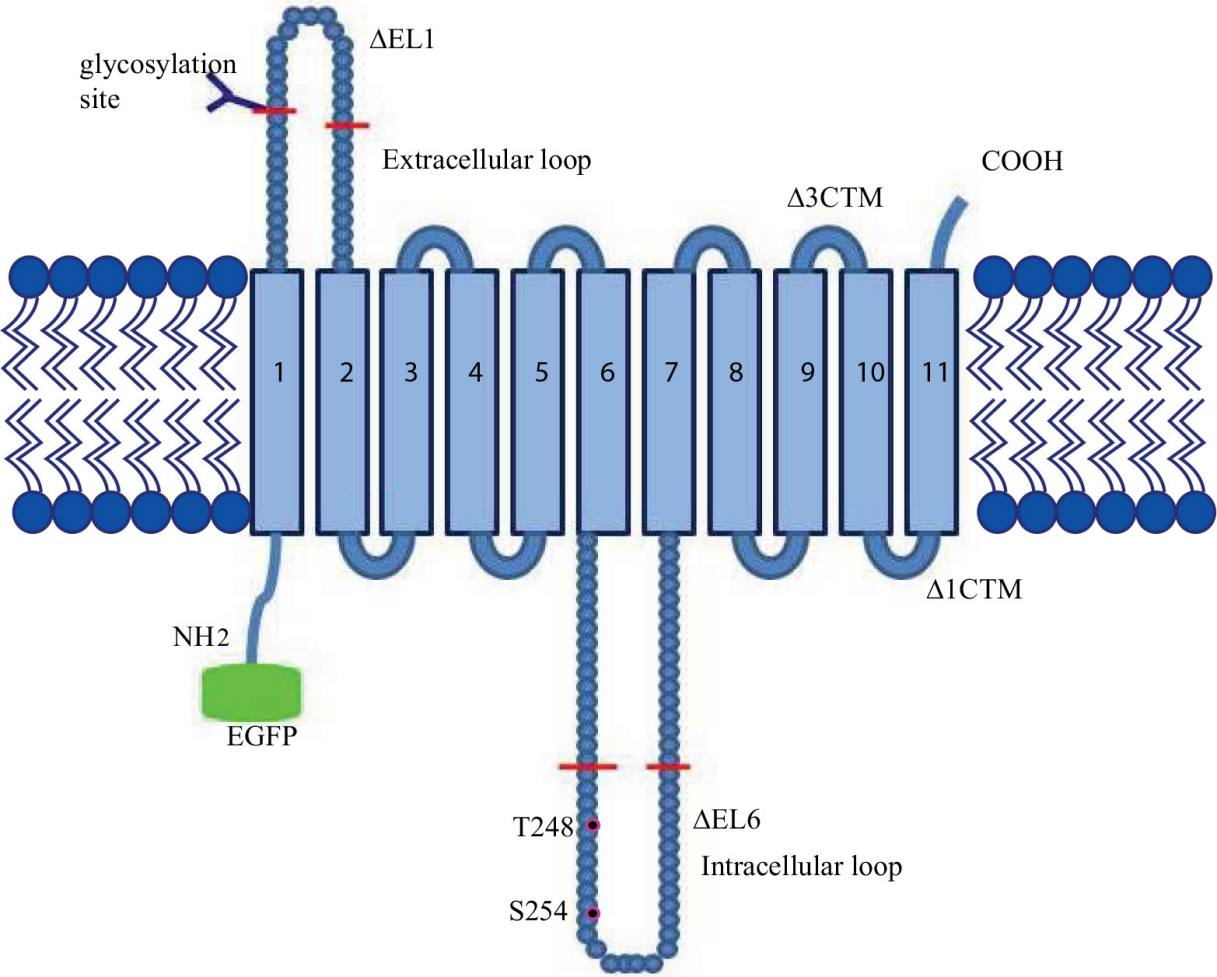
Structure of hENT1 with deletions and mutations. Shown is the predicted topology of hENT1 with the cytoplasmic amino-terminus, 11 TMs and short extracellular carboxyl-terminus. Red bars show the beginnings and the ends of the deleted regions in the extracellular, ∆EL1, and intracellular, ∆IL6, loops and the beginnings of the deletions at the carboxyl-terminus, ∆3CTM and ∆1CTM. The predicted CK2 phosphorylation sites, Thr 248 and Ser 254, are marked in magenta. EGFP was fused to the amino-terminus.

Equilibrative nucleoside transporters (ENTs) are integral membrane proteins, which facilitate passive diffusion of nucleosides and nucleobases across membranes [1]. Together with evolutionary and structurally unrelated sodium-dependent concentrative nucleoside transporters (CNTs), ENTs play key roles in nucleoside salvage for recycling in cells [2]. In addition, they regulate the physiological levels of adenosine, a major paracrine signaling molecule and neuromodulator [2,3]. Human ENTs comprise 4 members, designated as ENT1–4 [4–8]. All four transport adenosine but differ in their abilities to transport other nucleosides or nucleobases [1]. ENTs are of particular biomedical interest because they serve as major conduits for the cellular uptake of antiviral and anticancer drugs [6]. In particular, the expression levels of human ENT1 (hENT1) correlate with the success of gemcitabine chemotherapy in several types of cancer [9,10]. While the crystal structure of a bacterial CNT homologue [11] has provided information on the architecture and molecular mechanism of CNTs, no atomic resolution structure of an ENT has been reported to date. Computational approaches were used to produce ENT structural models based on crystal structures of major facilitator superfamily (MFS) transporters [12,13]. However, topological predictions suggest that ENTs comprise 11 trans-membrane segments (TMs) compared to typical 12 TM topology of MFS transporters. The amino- and carboxyl-termini of ENT are cytoplasmic and extracellular, respectively [14] (Fig 1). Large extra- and intra-cellular loops are located between TMs 1 and 2 (EL1) and TMs 6 and 7 (IL6).

In hENT1, these loops contain approximately 40 and 60 amino acids, respectively. The amino acid sequences of IL6 are highly variable and are predicted to be unstructured in solution. NMR studies of an isolated peptide corresponding to IL6 revealed an absence of detectable secondary structure [15]. However, the lack of intrinsic structure in IL6 does not preclude it from interacting with other proteins, which may regulate targeting and activity of the transporter. Furthermore, there are several charged residues within the loop that are conserved between organisms and impose polarity on the loop [15]. Similar unstructured and highly polar loops have been shown to modulate both the activity and proper folding in other transporters [16], potentially by stabilizing specific conformational states [17]. Moreover, several phosphorylation sites have been predicted within IL6 [18]. A predicted Casein Kinase II (CK2) phosphorylation site in IL6 of a mouse ENT1 orthologue was implicated in modulating the expression level of the transporter and its affinity for the inhibitor nitrobenzylthioinosine (NBMR), and CK2 played a role in activating hENT1 [19]. A splice variant lacking the site lost CK2-mediated regulation [20].

The only known glycosylation site of hENT1 Asn 48 is located in EL1 [14] (Fig 1). Glycosylation of hENT1 has been implicated in tight binding of NBMPR [21] and glycosylation sites in the analogous loop of hENT2 were essential for efficient targeting to plasma membrane [22]. In addition, EL1 of hENT1 also contains a mitochondrial localization motif [23]. Collectively, these results suggest that EL1 and IL6 may participate in protein targeting and modulate transport activity.

Several splice variants of the mammalian ENTs identified in recent years have distinct tissue distribution and sub-cellular localization [24–26]. These splice variants, lacking either one [24] or three [25] carboxyl terminal TMs, demonstrated altered substrate transport kinetics and sensitivity to inhibitors. These results suggested that the terminal TM segments of ENTs might play modulatory roles.

In this study, our aim was to define the role of the extracellular and cytoplasmic loop and the carboxyl terminus of hENT1 in stability, activity and targeting the transporter. Towards this end, hENT1 constructs with loop and carboxyl terminal deletions were fused to enhanced green fluorescent protein (EGFP) at their amino-termini and expressed in *Xenopus laevis* oocytes. Large deletions in the loops were very well tolerated and had only minor effects on the expression level of the transporter in the plasma membrane, or on the kinetic properties of hENT1-catalyzed nucleoside uptake and NBMPR binding. However, these deletion mutants were trafficked with diminished efficiency and showed increased accumulation throughout the secretory pathway. In contrast, deletions of the carboxyl terminal TM segments had deleterious effects on protein folding and targeting.

To determine whether CK2 phosphorylation plays a role in targeting hENT1 to the plasma membrane, residues at the CK2 predicted sites, Thr 248 and Ser 254 were mutated to alanine. Confocal images of these mutants showed normal trafficking, suggesting that the loss of CK2 sites had minimal effects on protein localization.

## Methods

### DNA cloning and expression in *Xenopus laevis* oocytes

Mutagenesis was performed using standard molecular biology techniques and all constructs were verified by DNA sequencing. Stage VI oocytes were obtained from wild type (WT) adult *Xenopus laevis* females (*Xenopus Express*) as described previously [27]. Briefly, ovaries were surgically removed after frogs were anesthetized in tricaine (5 g L^-1^), and digested enzymatically in collagenase type 1A (2 mg mL^-1^ in Ca^2+^-free Ringer solution) under gentle shaking to remove the follicular cells and then stored at 18°C. Oocytes were kept in 0.5 x L15 (*Sigma*). hENT1 sequences were sub-cloned into the *Xenopus* oocyte expression vector pSGEM, which flanks the cDNA with the 5’- and 3’-UTRs from the *Xenopus* globin gene thus stabilizing the resultant mRNA in the oocyte [28]. RNA was transcribed *in vitro* using mMESSAGEmMACHINE T7 Ultra Kit (*Ambion*). RNA integrity and concentration were validated on denaturing agarose gels before injection into oocytes. To minimize suffering, frogs were immersed in tricaine (1.5g/L, pH 7 with Na-bicarbonate) for 20 min before sacrificing. The depth of anesthesia was monitored by pinching the toe and monitoring the reaction. Once no reaction was registered the donor female was sacrificed by cranial pithing. Death was confirmed by lack of response to sharp foot pressure and by monitoring heart contractions. The protocol was approved by the Weill Cornell Medical Collage IACUC committee, Protocol # 2011–0035.

### Confocal imaging

To study membrane localization of the mutated ENTs, oocytes were co-injected with 23 ng of EGFP-hENT1 RNA and 13 ng of RNA encoding TMEM16a (plasma membrane marker) tagged with mCherry at the carboxyl terminus (mch-TMEM) [29]. Imaging was conducted using LSM 710 confocal microscope (*Zeiss*) using EC Plan Neofluar 40x/1.30 oil DIC M27 objective. Oocytes were scanned in Ringer solution, containing in mM 5 HEPES, pH 7.4, 125 NaCl, 3 KCl, 1.8 CaCl_2_, 10 glucose and 1.6 MgCl_2_. Image acquisition and analysis was performed using ZEN 2008 software (*Zeiss*). To study the intracellular distribution of hENT1 proteins, oocytes were co-injected with 23 ng of EGFP-hENT1 RNA and 18 ng of RNA encoding KDEL (ER marker) fused to mCherry (mch-KDL). Oocytes were imaged as above. An entire z-stack of images was collected on each oocyte starting at the plasma membrane focal plane and going deep within the oocyte. During analysis, a region of 312 x 312 μm was chosen and intensity of EGFP on the membrane and in ER was calculated as a fraction of the total fluorescence. Figures were compiled using Adobe Illustrator (*Adobe*). The values are expressed as mean ± SE. The results were computed statistically (SPSS software package, version 20) using one-way analysis of variance (ANOVA). Post hoc testing was performed for inter-group comparison using Student-Newman-Kuel multiple comparison test. Values of p< 0.05 were considered significant.

### Nucleoside uptake assays

Transport assays were performed as described previously [6]. In brief, batches of 5 oocytes were incubated at room temperature in 200 μl of transport buffer containing in mM, 100 NaCl, 2 KCl, 1 CaCl_2_, 1 MgCl_2_ and 10 HEPES, pH 7.5 in the presence of ^14^C-adenosine (0.1 mCi ml^-1^), ^14^C-uridine (0.1 mCi ml^-1^) or ^3^H NBMPR (0.1 mCi ml^-1^) (*Moravek Biochemicals*). For all experiments, the oocytes were pre-incubated in transport buffer for 15 min before addition of the substrates. The time-dependent uptake studies for adenosine and uridine were carried out for 1–60 min after addition of 5 μM substrates. Concentration dependencies were determined by incubating oocytes with adenosine and uridine at concentrations between 10 μM and 5 mM for 1 and 3 minutes, respectively. Following incubation, oocytes were washed 6 times in 1 ml ice-cold transport buffer to remove the unbound substrate, lysed in 200 μl of 1% SDS and quantified using a liquid scintillation counter (*Perkin Elmer*) after addition of 2 ml scintillation fluid. Results for the transport assays are given as means ± SE for 5 oocytes.

### Western blot

For western blots, oocytes were lysed by pipetting up and down in buffer containing in mM 30 HEPES, pH 7.5, 100 NaCl, 100 NaF, 2 mM sodium vanadate, 50 β-glycerolphosphate, 10 sodium phosphate, 5 EDTA, 5 EGTA and 1 DTT and centrifuged twice at 1000 rcf to remove debris. The supernatant was treated with 20 mM decyl β-D-maltopyranoside (*Sigma*) for two hours. Solubilized membranes were centrifuged at 12,700 rcf for 20 min and the supernatant was analyzed by SDS-PAGE (*BioRad*). Proteins were transferred to nitrocellulose membrane, blocked with 5% milk and probed with monoclonal anti-GFP antibody (*Clontech*). The membranes were further incubated with peroxidase-labeled secondary antibody (*Sigma*) in 5% milk and the protein bands were visualized using the Chemiluminescent Substrate kit (*Pierce*). Actin (*Sigma*) was used as the internal control. To deglycosylate the WT hENT1 and ∆IL6, 100 μg of the proteins were incubated with 10 μl of the deglycosylase mix (*NEB*) at 37°C and analyzed by SDS-PAGE. Each experiment was performed in triplicate on different batches of oocytes. The bands were quantified using gel documentation system (*Perkin Elmer*) and expressed as percentage of the control.

### NBMPR binding

To determine the number of the substrate binding sites, oocytes were injected with hENT1 RNA as above, incubated with 500 nM ^3^H NBMPR for 1 hr, washed and the retained radioactivity was quantified. To obtain NBMPR inhibition constants, injected oocytes were incubated with 0.5–500 nM NBMPR (*Sigma*) for one hour, and the uptake of 5 μM of ^14^C-adenosine was measured after 1 min incubation. Retained radioactivity was quantified as above.

### CK2 inhibition by tetrabromobenzo-triazole (TBB)

To determine the effect of TBB on hENT1, the injected oocytes were incubated with 25 μM TBB for 12 hours and assayed for ^14^C adenosine uptake. Confocal images were taken after co-injection of the RNA encoding EGFP-hENT1 and plasma membrane marker *Xenopus* TMEM16A gene tagged with mCherry (mch-TMEM) or ER marker mCherry-KDEL (mch-KDEL) as described above. Protein levels were determined by western blotting.

## Results

### hENT1 and loop deletions localize to the plasma membrane

**Fig 2:**
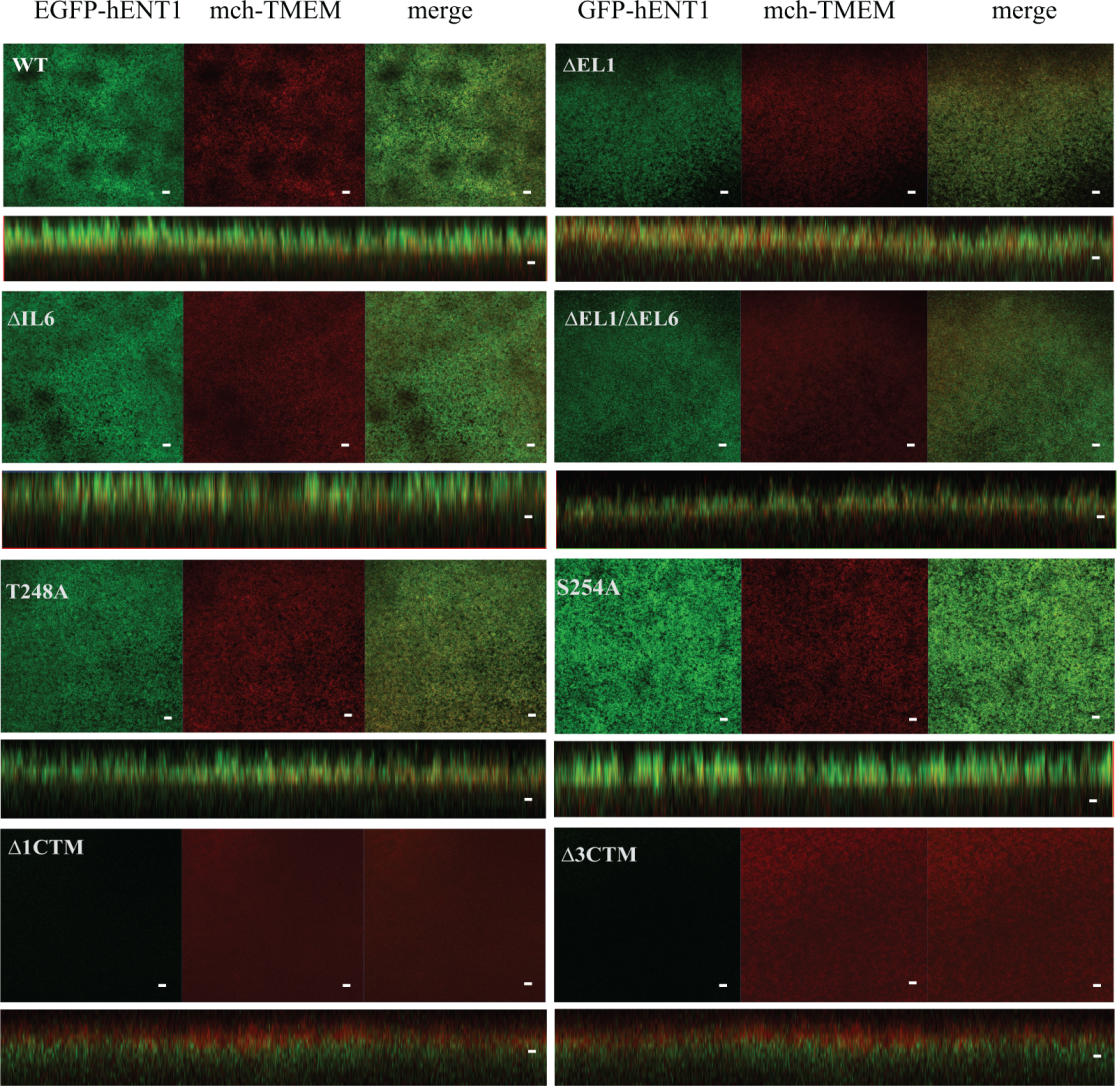
Plasma membrane localization of hENT1 variants. Fluorescent confocal microscope images of WT hENT1, ∆EL1, ∆IL6, and ∆EL1/∆IL6 loop deletion mutants, T248 and S254A point mutants, and ∆1CTM and ∆3CTM carboxyl-terminus deletion mutant expressed in *X*. *laevis* oocytes. Constructs are indicated on the panels. The individual images along the focal plane of the microvilli on the membrane (EGFP in green, mch-TMEM in red) are shown, followed by the merge of EGFP and mch-TMEM in (yellow). Orthogonal sections through Z stacks also show co-localization. Carboxyl-terminal deletions did not show EGFP fluorescence on the membrane. Images are representative of 30 oocytes. Scale bar = 5μm.

To probe the role of EL1 and IL6 in hENT1 targeting to plasma membrane, mutants were generated, in which residues Asn 48 to Ala 65 in EL1 (∆EL1, deletion mutant in extracellular loop 1) and Pro 243 to Gln 274 in IL6 (∆IL6, deletion mutant in extracellular loop 6) were deleted (Fig 1). The WT hENT1 and the deletion constructs were tagged with EGFP at their amino-termini. RNAs encoding the EGFP-hENT1 fusion constructs were co-injected with a plasma membrane marker mch-TMEM, used as a marker of plasma membrane localization. Confocal fluorescence microscopy revealed that WT EGFP-hENT1 co-localized with mch-TMEM to plasma membrane, confirming that hENT1 is targeted correctly. The loop deletion mutants, ∆EL1, ∆IL6 and the double deletion mutant ∆EL1/∆IL6 were also targeted to the plasma membrane (Fig 2). Therefore, neither glycosylation of Asn 48 in EL1 nor phosphorylation at the predicted CK2 sites in IL6 is required for the correct targeting of hENT1. Similar results were also obtained when the predicted CK2 sites in IL6, Thr 248 and Ser 254, were mutated to alanine (Fig 2). In contrast to the loop deletions, the carboxyl-terminal deletion mutants, lacking either 100 (∆3CTM, deletion mutant lacking TM9-11) or 32 (∆1CTM, deletion mutant lacking TM11) residues, corresponding to the last 3 and 1 TMs respectively, showed no plasma membrane localization (Fig 2). Instead, as revealed in the orthogonal section across the z-stack, both deletion mutants localize deep within the cell away from the cell membrane marked by mch-TMEM (Fig 2).

### Intra- and extra-cellular loops facilitate efficient trafficking of hENT1

**Fig 3:**
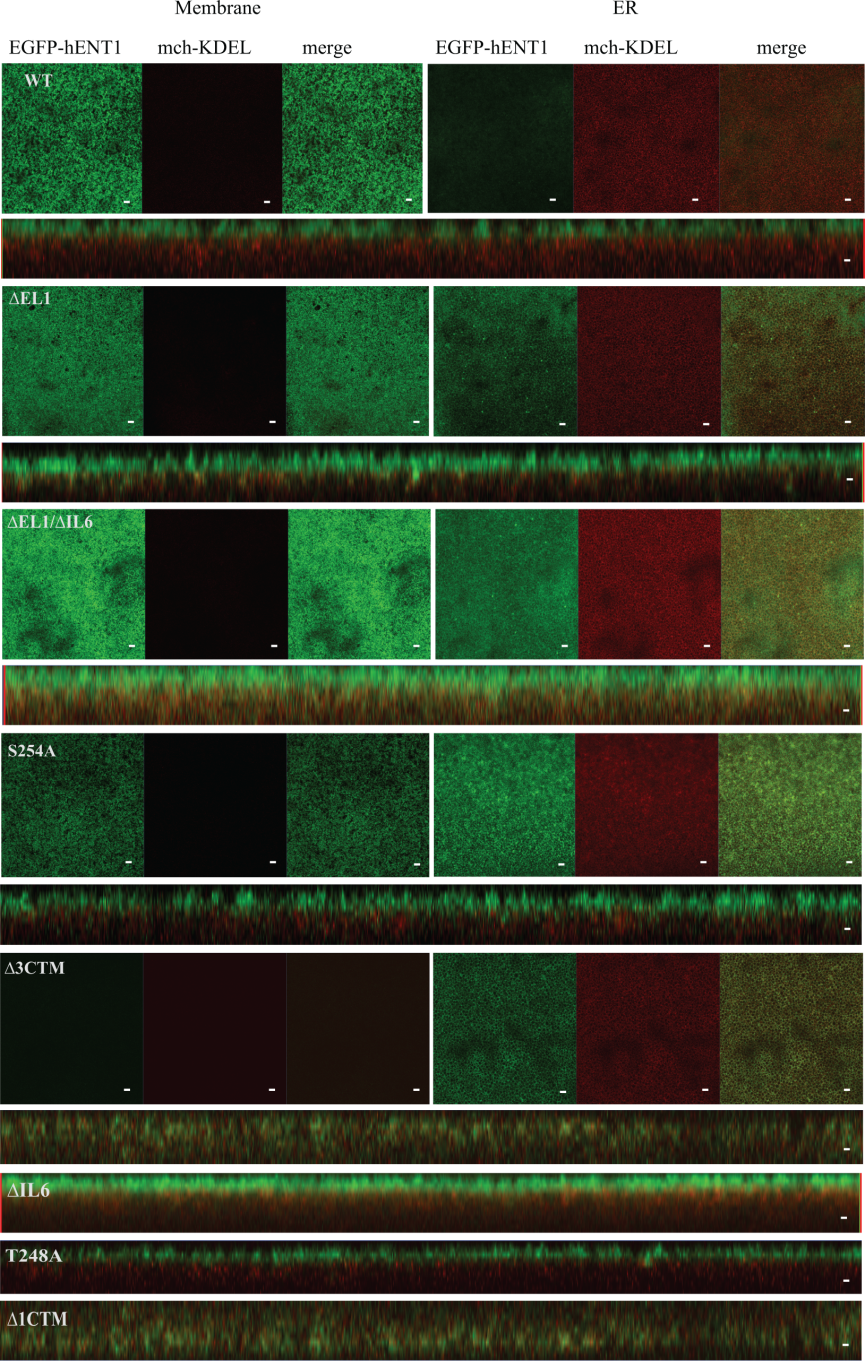
Intracellular distribution of the hENT1 variants. Shown are the confocal images for WT hENT1, ∆EL1, and ∆EL1/∆IL6 loop deletion mutants, S254A point mutant, ∆3CTM carboxyl-terminus deletion mutant expressed in *X*. *laevis* oocytes. Two images were taken: one on the plasma membrane (left panel) and the other deep within the cell showing the ER structure (right panel). Only the orthogonal sections are shown for ∆IL6, T248A and ∆1CTM. Left panel shows normal distribution of EGFP (green) on the membrane and mch-KDEL (red) in the ER for the WT, T248A and S254A. In contrast, ∆EL1, ∆IL6, ∆EL1/∆IL6 shows some co-localization of hENT1 and KDEL in the ER. Carboxyl-terminal deletions did not show EGFP fluorescence on the membrane. Instead, EGFP was present within the ER, co-localized with mch-KDEL. All the confocal images show a single, representative, section of a Z-series taken through the entire cell. Images are representative of 30 oocytes. Scale bar = 5μm.

Newly synthesized polypeptides that fail to fold properly, are retained within the ER and destined for degradation [30]. In order to establish whether the soluble loops are important for efficient protein folding and ER exit, EGFP-hENT1 mutant RNAs were co-injected with an ER marker mch-KDEL. Confocal imaging revealed enrichment of EGFP-hENT1 at the plasma membrane plane, with little localization to the ER for the WT protein. In contrast, ∆EL1 and ∆IL6 deletion mutants showed substantially higher intracellular localization, likely in the ER (Fig 3). The CK2 mutants behaved like the WT, showing EGFP fluorescence predominantly at the plasma membrane, and mch-KDEL fluorescence in the ER (Fig 3). As expected, the carboxyl-terminal deletion mutants were fully retained within the ER, co-localizing with mch-KDEL (Fig 3), suggesting that the proteins lacking these regions did not achieve native-like structure and did not progress through the secretory pathway. Only the orthogonal sections are shown for ∆IL6, T248A and ∆1CTM.

**Fig 4:**
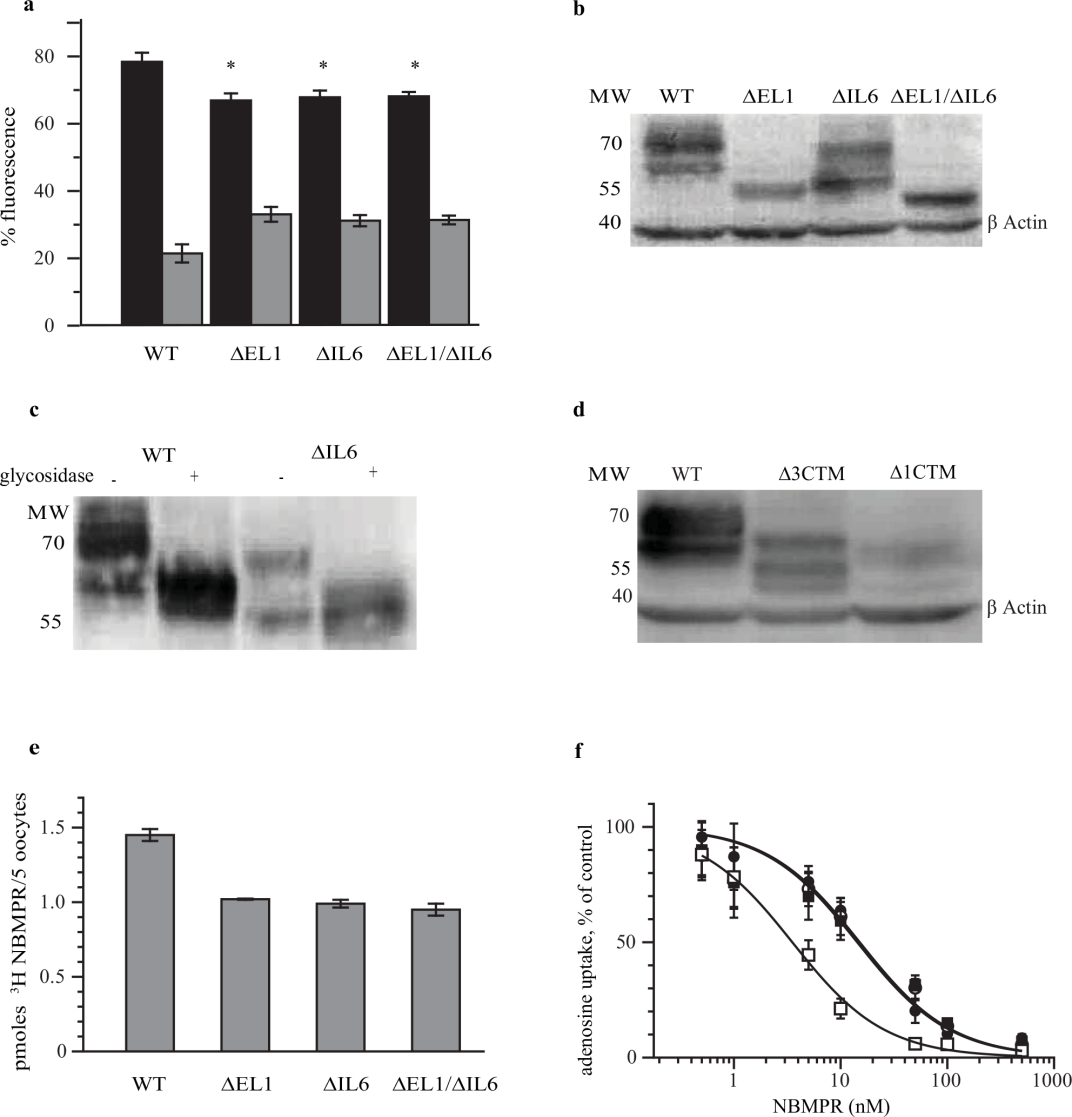
hENT1 loop deletion mutants show similar sub-cellular distribution and susceptibility to NBMPR inhibition. (a) Fraction of intra- and extra-cellular distribution of EGFP-hENT1 variants. Confocal images shown in Fig 2 were quantified and the fluorescence in the ER and plasma membrane expressed as percent of the total fluorescence. Shown are means and SE for 30 oocytes. * p<0.05. The black and grey bars represent the extra and intracellular fluorescence respectively. (b) The total protein expression assessed by Western blot. 100 μg of proteins were loaded in each lane. The EGFP-hENT1 fusion proteins were visualized using EGFP monoclonal antibody. Constructs are shown above the lanes. (c) Western blots showing deglycosylation of the WT and ∆IL6 mutant. 100 μg of the proteins were incubated with and without deglycosylase for 4 hr at 37°C. (d) Western blots for carboxyl terminal deletions. 400 μg of the WT, ∆3CTM and ∆1CTM deletions were loaded in each lane and visualized with EGFP monoclonal antibody. The migration positions of the molecular weights standards are indicated in panel’s b-d. Actin was used as the internal control to show equal loading in lanes b and d. (e) Quantification of NBMPR binding sites. Oocytes were injected EGFP-hENT1 RNA were incubated for 48 hrs prior to addition of 500 nM ^3^H NBMPR for one hr. Radioactivity retained by washed oocytes was measured and corrected for background. Values are expressed as mean ± SE for 5 oocytes for 3 independent experiments done in triplicate. (f) Adenosine uptake after NBMPR inhibition. Values corrected for background are expressed as mean ± SE for 5 oocytes for 3 independent experiments done in triplicate. Shown are WT (closed circles), ∆IL1 (open circles), ∆IL6 (closed squares), and ∆EL1/∆IL6 (open squares).

Quantitative analysis of the confocal images showed that ~30% of the loop deletion mutants were retained in the ER compared to ~20% of the WT protein (Fig 4A). These differences are statistically significant, suggesting that the loops are involved in exporting hENT1 out of the ER, and possibly facilitate efficient protein folding. The overall expression levels of the WT and hENT1 loop deletion mutants were quantified by western blot using monoclonal hENT1 antibody. The WT protein and ∆IL6 mutant migrated as double bands, while the ∆EL1 and ∆EL1/∆IL6 mutants showed only single bands (Fig 4B). It is likely that the lower and higher molecular weight bands of the WT hENT1 and ∆IL6 mutant are due to the unglycosylated and glycosylated forms of the proteins, respectively. Consistently, enzymatic de-glycosylation of the proteins prior to SDS PAGE eliminated the higher molecular weight bands (Fig 4C). Quantification of the bands shows that the total protein decreased by 50 ± 4.4, 63 ± 18 and 62 ± 12% for ∆EL1, ∆IL6 and ∆EL1/∆IL6, respectively, when compared to the WT. For the carboxyl-terminal deletion mutants, we observed multiple bands in the Western blots showing that the proteins underwent degradation (Fig 4D).

To quantify the amount of transporters expressed on the cell surface, binding of ^3^H-NBMPR to the WT protein and loop deletion mutants were measured. The amount of NBMPR bound was ~30% lower for the deletion mutants compared to the WT protein (Fig 4E). Since NBMPR is a competitive inhibitor of the transporter, the amount of ^3^H-NBMPR bound to cells reflects the total amount of hENT1 protein expressed on the surface, assuming that hENT1 does not recycle significantly during 1 hour long NBMPR incubation period. If hENT1 is recycled then NBMPR is expected to label the entire hENT1 pool. However, two pieces of evidence argue that hENT1 in oocytes does not recycle significantly. First, EGFP-hENT1 does not exhibit any staining of the endosomal compartments, which would be expected for a recycling protein. Second, the results obtained with NBMPR labeling a consistent with those obtained from confocal imaging (Fig 4A), which also show ~30% reduction in surface expression for the loop deletion mutants.

### The soluble loops are not required for transport activity

**Fig 5:**
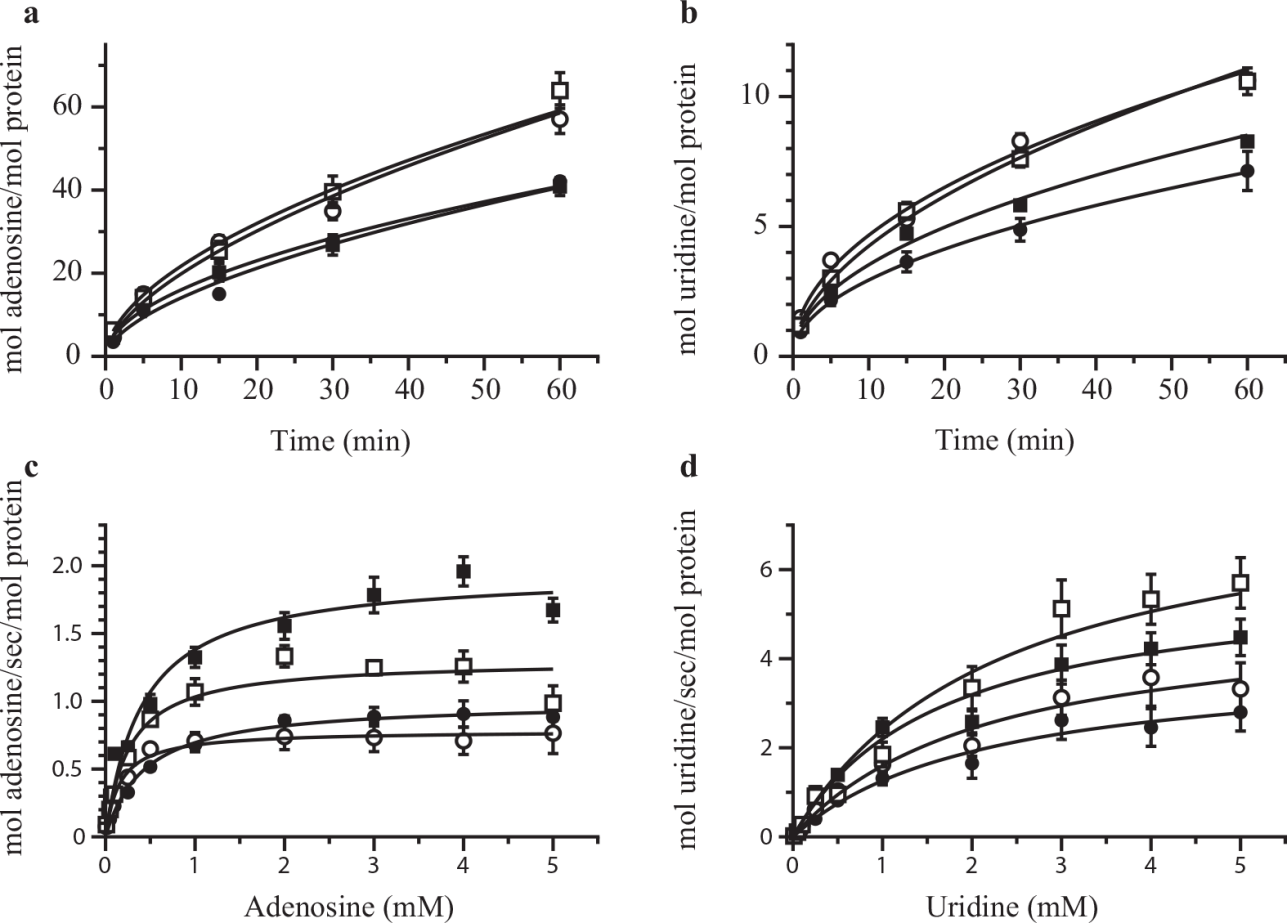
hENT1 deletion mutants show similar substrate uptake. Time dependence of uptake of 5 μM adenosine (a) and uridine (b). Concentration dependence of adenosine (c) and uridine (d) uptake. The incubation time was 1 minute for adenosine and 3 minutes for uridine. Values are corrected for background and expressed as means ± SE for 5 oocytes for 3 independent experiments done in triplicate. Shown are WT (closed circles), ∆EL1 (open circles), ∆IL6 (closed squares), and ∆EL1/∆IL6 (open squares).

The low endogenous nucleoside transport in *X*. *laevis* oocytes makes them well suited to assess transport of nucleosides and nucleoside analogs mediated by recombinant CNTs and ENTs [31,32]. Therefore this model system was used to ascertain the functionality of the WT hENT1 and the deletion mutants. The uptake of both adenosine and uridine remained mostly linear for ~10 minutes (Fig 5A and 5B), declining modestly at longer times. Continuous uptake at even longer time might be due to the cellular metabolism of the substrates, which effectively reduces their intracellular concentration. No significant differences were observed between the WT and the loop deletion mutants with initial rates ranging between 2 and 4 mol substrate/min/mol protein for adenosine and between 0.4 and 0.8 mol substrate/min/mol protein for uridine. The *Km* and *Bmax* were also similar between the constructs (Fig 5C and 5D and Table 1). We observed some variability between constructs in *Km* and *Bmax* values for adenosine uptake. However, the significance of these differences is not clear because they were not paralleled by similar differences in uridine uptake. Collectively, these data show that the deleted regions are not necessary for the transport activity, and if they do modulate transport, their effects are subtle. The uptake of adenosine by WT hENT1 and loop deletion mutants was inhibited by NBMPR with *IC50* similar between the constructs (Fig 4F and Table 1). While we observed a more efficient inhibition of the ∆EL1/∆IL6 mutant, the observed difference of about 4 fold would require further investigation to allow mechanistic interpretation. We note that our results differ from a previous observation that mutation of the glycosylation site in the first extracellular loop leads to decreased affinity of hENT transporter expressed in *Saccharomyces cerevisiae* for NBMPR [21]. We did not observe reduced NBMPR sensitivity in ∆EL1 and ∆EL1/∆IL6 constructs that lack the glycosylation site. This might be because hENT1 expressed in oocytes and in yeast undergo distinct glycosylated.

**Table 1:** The kinetic analysis of transport by hENT1 constructs.

	WT	∆EL1	∆IL6	∆EL1/∆IL6
*K*_*m*_, adenosine	440 ± 50 *	160 ± 20	410 ± 90	262 ± 70
*B*_*max*_, adenosine	1.0 ± 0.03	0.78 ± 0.02	2.0 ± 0.1	1.3 ± 0.08
*K*_*m*_, uridine	2,160 ± 500	2,180 ± 540	1,670 ± 380	2,520 ± 190
*B*_*max*_, uridine	4 ± 0.3	5.1 ± 0.5	5.8 ± 0.5	8.3^#^
*IC50*, NBMPR	15 ± 3	15 ± 3	14 ± 3	3.5 ± 0.3**

*The *Km*, *Bmax* and *IC50* values are in μM, mol substrate/sec/mol protein, and nM, respectively.

^#^The parameter could not be unambiguously fitted and was fixed at the reported value. The values were obtained by fitting data in Figs 4 and 5.

** Comparison between the WT and ∆EL1/∆IL6. P<0.05.

### The effects of TBB on the expression level, localization and glycosylation of hENT1 constructs

**Fig 6:**
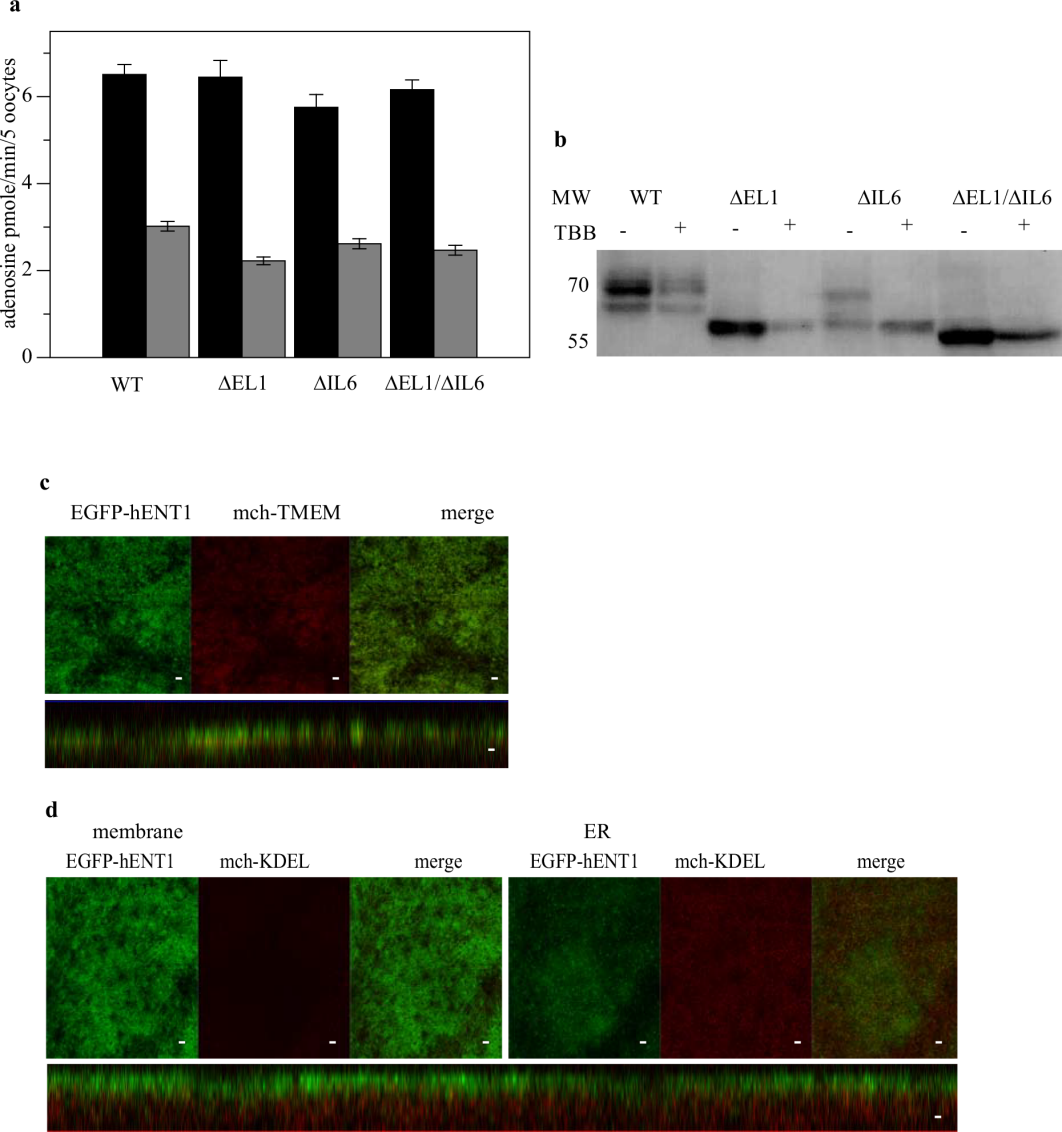
Effect of TBB on the deletion constructs. (a) Uptake of 5 μM ^14^C-adenosine after 1 minute incubation period without (black) and with (gray) pre-incubation with TBB. Values are expressed as mean ± SE for 5 oocytes for 3 independent experiments done in triplicate. (b) Western blot of the oocytes with and without treatment with TBB probed with EGFP monoclonal antibody. (c) Confocal images of oocytes co-injected with RNA expressing WT EGFP-hENT1 and mch-TMEM after treatment with 25 μM TBB for 12 hr. (d) Confocal images of oocytes co-injected with RNA expressing WT EGFP-hENT1 and mch-KDEL. Images were taken along the plane of the microvilli (left) and within the ER (right).

We observed that mutations (T248A and S454A) of the proposed CK2 phosphorylation sites did not have major effects on trafficking and surface expression of hENT1. In order to see the effect of CK2 on the sorting and expression level of hENT1 and its mutants, inhibition by TBB was carried out. Incubation of oocytes expressing WT hENT1 and the loop deletion mutants for 12 hrs with 25 μM TBB led to inhibition of adenosine transport by over 50% in all constructs (Fig 6A). Consistently, the overall expression of the transporter decreased after treatment with TBB (Fig 6B). Interestingly, there was a complete loss of the glycosylated band for ∆IL6 but not the WT protein. Confocal images showed that the cellular architecture was altered following TBB treatment and revealed an uneven protein distribution on the plasma membrane. Though WT EGFP-hENT1 was still correctly targeted to the plasma membrane, the amount of EGFP-hENT1 protein retained within the ER increased showing co-localization with mch-KDEL (Fig 6C and 6D).

## Discussion

Approximately 30–60% of eukaryotic proteins are predicted to contain intrinsically disordered regions [33],[34]. Specifically, over 40% of plasma membrane proteins contain such regions with much higher occurrence on the intracellular compared to the extracellular side [35]. The intracellular disordered regions have been experimentally demonstrated in hENT1 [15], the sodium hydrogen exchanger, and the voltage-gated potassium channel [36,37]. In the latter two proteins, these loops were shown to participate in protein/protein interactions and play important roles in protein targeting. An extracellular loop of sodium/bicarbonate symporter, thought to be unstructured, has recently been shown to play an important role in determining the stoichiometry of transport [38].

Here, we aimed to examine the role of unstructured loops in folding, trafficking, targeting and activity of hENT1. In these studies we deleted large portions of the loops, and generated mutants, targeting potential phosphorylation sites. The co-localization of the plasma-membrane marker mch-TMEM with EGFP-hENT1 as well as with loop deletion constructs show that all of these proteins are correctly folded and targeted. These results suggest that the unstructured loops are not necessary for the correct protein folding and do not play a major role in determining the localization of hENT1. Interestingly, our results show that the correct trafficking is achieved even in the absence of the glycosylation site Asn 48.

Although our results suggest that the soluble loops are not required for folding or plasma membrane targeting of hENT1, they may contribute to the efficiency of these processes. Indeed, the loop deletion mutants were expressed at lower levels compared to the WT, both overall and at the plasma membrane. They were also retained in the ER to a greater extent, as they showed more efficient co-localization with the ER membrane marker mch-KDEL. Interestingly, deletions within either the extracellular or the intracellular loop had similar effects. Whether these loops contribute to more efficient folding, or participate in interaction with other proteins remains unknown. Notably, we expressed hENT1 protein heterologously in X. *laevis* oocytes, where native interaction partners of hENT1 are absent. Nevertheless, our results suggest that with or without the loops, hENT1 can reach its final plasma membrane destination.

The kinetic measurements suggest that the extracellular and intracellular loops do not play a significant role in modulating the affinity of the transporter for its substrates, adenosine and uridine, or for the inhibitor NBMPR. Furthermore, similar maximum uptake rates for all constructs were observed. Considering that the expression level of the loop deletion mutants was lower than the WT, these results may indicate that the presence of the loops mildly inhibits transport. However, further experiments would be needed to confirm these moderate effects. Overall, we conclude that the soluble, likely unstructured, extracellular and intracellular loops play little role in the transport activity of the protein.

Neither the deletion nor mutation of potential CK2 phosphorylation sites in IL6 had a major effect on protein folding or targeting, showing that phosphorylation is not required for these processes. Addition of TBB, an inhibitor for CK2, affected the architecture of the plasma membrane, increased protein degradation, decreasing the overall expression level of the WT and mutant transporters. These effects were confirmed by the confocal images and western blots. Intriguingly, TBB completely abolished glycosylation of the ∆IL6 mutant but not the WT. Further experiments are required to establish the underlying mechanism of these observed differences. The decrease of the overall protein expression was on par with the decrease in adenosine transport. Since ∆IL6, which lacks the CK2 sites, is also affected by TBB, the effect of the CK2 inhibitor must be indirect and not due to CK2 acting on hENT1. It is possible that CK2-dependent regulation of hENT1 is not observed in our experiments because we use a heterologous expression system lacking the necessary regulatory components. Interestingly, an earlier report on mouse ENT1 lacking the CK2 consensus site also suggests that ENT1 might be regulated by other mechanisms [39].

Previously, shorter splice variants have been identified that lacked the last 1 and 3 carboxyl terminal TMs in rabbit and mouse ENT1, respectively [24, 25]. These splice variants have been reported to be functional, albeit with altered kinetic properties. Here we examined the effects of analogous deletions at the carboxyl terminus of hENT1. Our experiments clearly show that deletions of these regions resulted in misfolded proteins that were retained in ER and underwent proteolytic degradation. These results suggest that the last three TMs are indispensable regions of the transporter’s structure. These results are consistent with the substantial residue conservation within these regions [40,41]. The reason for differences with earlier studies on splice variants is unclear at this time. However, intracellular retention of hENT1 lacking 156 carboxyl terminal residues has also been previously reported [26].
